#  Restoration and coral adaptation delay, but do not prevent, climate-driven reef framework erosion of an inshore site in the Florida Keys

**DOI:** 10.1038/s41598-022-26930-4

**Published:** 2023-01-05

**Authors:** Alice E. Webb, Ian C. Enochs, Ruben van Hooidonk, René M. van Westen, Nicole Besemer, Graham Kolodziej, T. Shay Viehman, Derek P. Manzello

**Affiliations:** 1 Ocean Chemistry and Ecosystems Division, NOAA Atlantic Oceanographic and Meteorological Laboratory, 4301 Rickenbacker Causeway, Miami, FL 33149 USA; 2 Rosenstiel School of Marine and Atmospheric Science, Cooperative Institute for Marine and Atmospheric Studies, University of Miami, 4600 Rickenbacker Causeway, Miami, FL 33149 USA; 3 Institute for Marine and Atmospheric Research Utrecht, Utrecht University, Princetonplein 5, 3584 CC Utrecht, The Netherlands; 4 National Centers for Coastal Ocean Science, NOAA National Ocean Service, 101 Pivers Island Rd, Beaufort, NC 28516 USA; 5 Coral Reef Watch, Center for Satellite Applications and Research, NOAA Satellite Oceanography and Climatology Division, College Park, MD 20740 USA

**Keywords:** Climate sciences, Ecology

## Abstract

For reef framework to persist, calcium carbonate production by corals and other calcifiers needs to outpace loss due to physical, chemical, and biological erosion. This balance is both delicate and dynamic and is currently threatened by the effects of ocean warming and acidification. Although the protection and recovery of ecosystem functions are at the center of most restoration and conservation programs, decision makers are limited by the lack of predictive tools to forecast habitat persistence under different emission scenarios. To address this, we developed a modelling approach, based on carbonate budgets, that ties species-specific responses to site-specific global change using the latest generation of climate models projections (CMIP6). We applied this model to Cheeca Rocks, an outlier in the Florida Keys in terms of high coral cover, and explored the outcomes of restoration targets scheduled in the coming 20 years at this site by the Mission: Iconic Reefs restoration initiative. Additionally, we examined the potential effects of coral thermal adaptation by increasing the bleaching threshold by 0.25, 0.5, 1 and 2˚C. Regardless of coral adaptative capacity or restoration, net carbonate production at Cheeca Rocks declines heavily once the threshold for the onset of annual severe bleaching is reached. The switch from net accretion to net erosion, however, is significantly delayed by mitigation and adaptation. The maintenance of framework accretion until 2100 and beyond is possible under a decreased emission scenario coupled with thermal adaptation above 0.5˚C. Although restoration initiatives increase reef accretion estimates, Cheeca Rocks will only be able to keep pace with future sea-level rise in a world where anthropogenic CO_2_ emissions are reduced. Present results, however, attest to the potential of restoration interventions combined with increases in coral thermal tolerance to delay the onset of mass bleaching mortalities, possibly in time for a low-carbon economy to be implemented and complementary mitigation measures to become effective.

## Introduction

The ability of coral reefs to maintain complex three-dimensional frameworks and to retain the potential for vertical accretion through time is crucial for the persistence of their ecological functions and the anthropocentric services they provide^1–3^. In addition to supporting an extraordinarily rich diversity of organisms^4,5^, the heterogeneous calcium carbonate (CaCO_3_) architecture of reefs reduces wave energy and protects the shoreline from erosion and flooding^6,7^. This reef structure is the result of carbonate accumulation where calcification by corals and other organisms exceeds loss via both biological and abiotic (physical and chemical) erosion^3,8^.

The balance between these processes is currently threatened by the multifarious effects of ocean warming and acidification^9–11^. Coral mortality from bleaching and disease, as well as depressed calcification from thermal stress and decreasing carbonate saturation state, are of particular concern because even small reduction in building capacity can tip reefs from net accretion into a state of net erosion^12,13^. Further, bioerosion is projected to increase as acidification favors the dissolution of CaCO_3_^14,15^.

The summation of all processes contributing to calcification and bioerosion on a reef, termed carbonate budget modelling, and the resultant potential vertical accretion can serve as useful metrics to gauge the capacity of a reef to maintain their structurally complex framework and to keep pace with sea-level rise (SLR)^3,13^. Carbonate budgets can be assessed by means of the *ReefBudget* approach^16^, a field-based census methodology based on the abundance of all major carbonate producing and eroding taxa, coupled with literature-supported contemporary rates at which these processes operate. This method offers an effective and convenient tool to assess budgets across biogeographic regions and time^17,18^. Multiple studies spanning the Caribbean and the Florida Keys have shown that many reefs are already net erosional^19,20^.

Although the protection and recovery of ecosystem function, such as coral growth and calcification, are the focus of most restoration and conservation programs (through coral planting), there is some debate whether reef restoration will continue to be successful under increasingly more severe stress and more frequent disturbances^21^. Decision makers are limited by the lack of predictive tools assessing regional responses under future climate change and evaluating the potential impact of local initiatives to mitigate effects of ocean acidification (OA) and warming^22^. Projecting trajectories of future net carbonate production is, however, challenging because the manifestation of the effects of OA and warming will not occur uniformly in space and time across the world’s reef regions^23–25^. Many different regional effects will impact temperature and aragonite saturation state (Ω_Ar_) differently^26,27^. For instance, effects from the El Niño-Southern Oscillation (ENSO), polar amplification and the slowdown of the Atlantic Meridional Overturning Circulation (AMOC) cause sea surface temperature rise to vary greatly across regions under climate change^28^. This adds to the uncertainties regarding the onset and severity of annual bleaching due to marine heatwaves^26^. Further, there is great variability in the calcification responses to warming and OA among different coral species, and the flexibility in the ability of some corals to maintain calcification rates over a broad range of Ω_Ar_ conditions^29–31^. The high spatial variation in rates of environmental change combined with species-specific ecophysiologies make projecting declines in coral cover and their contribution to net carbonate production difficult for any world region or reef habitat type^27,32,33^.

To address this environmental and biological-specificity, we build tailored carbonate budget projections that tie species-specific responses to site-specific global change using the latest generation of climate models projections (CMIP6)^28^. We apply our modeling approach to Cheeca Rocks, an outlier reef in the Florida Keys in terms of high coral cover, carbonate production and abundance of *Orbicella faveolata*, which has suffered considerable loss across the wider region. This site is one of the three National Coral Reef Monitoring Program (NCRMP) sentinel sites for climate and ocean acidification monitoring, as well as one of the seven reefs targeted in the Mission: Iconic Reefs (M:IR) restoration initiative.

We examine the impact of different greenhouse gas emission scenarios on carbonate production, accretion potential, and the capacity of the reef to keep up with SLR under various thermal adaptive levels in corals. Finally, the impact of M:IR restoration targets are assessed in the context of warming temperatures and increased acidification at this site, as well as with incorporated thermal adaptation in restored corals.

## Results and discussion

**Figure 1:**
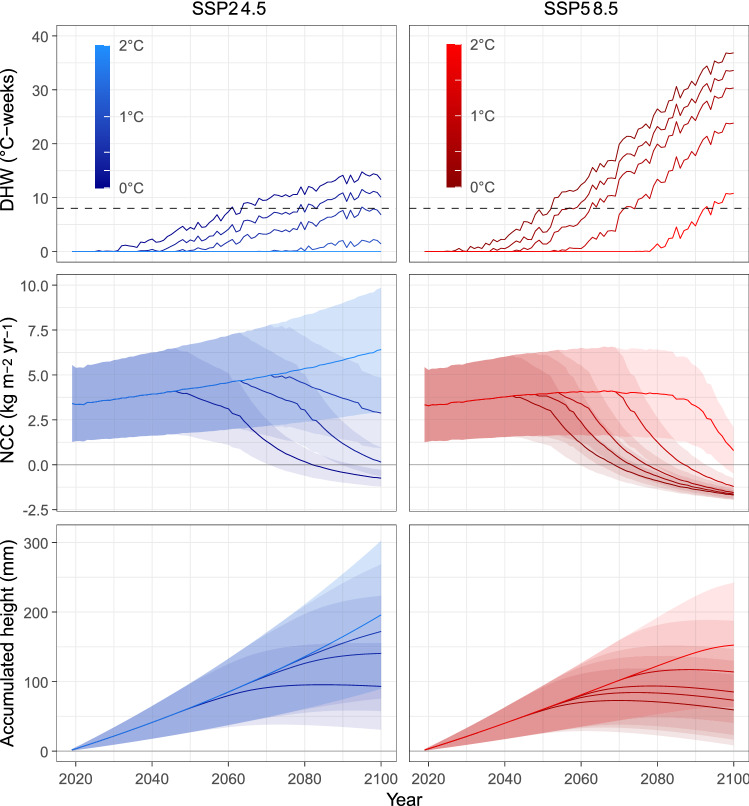
Trajectories of degree heating weeks, net carbonate budgets, and accumulated reef height at Cheeca Rocks under two CO_2_ emission scenarios and across five thermal adaptation levels. The top two panels represent Degree Heating Weeks (DHWs; see methods 3.2) calculated while increasing the bleaching threshold by 0, 0.25, 0.5, 1 and 2˚C under the SSP2-4.5 (in blue) and SSP5-8.5 (in red) scenarios. The dashed line represents the onset threshold (DHW = 8) of annual severe bleaching. The middle panels illustrate the projected mean net community calcification (in kg m^−2^ yr^−1^) across the six transects at Cheeca Rocks. The blue and red shaded areas depict the standard deviation from the mean for the SSP2-4.5 and the SSP5-8.5 scenarios respectively and the horizontal grey lines represent the threshold (NCC = 0) when carbonate budgets become negative. The bottom panels depict the mean accumulated height calculated from the sum of past Reef Accretion Potential (RAP_max_; see methods 3.3). The horizontal grey line represents the present reef height.

Regardless of emission scenarios, coral adaptive capacity, or restoration, trajectories of modelled net carbonate production at Cheeca Rocks decline heavily once the threshold for the onset of annual severe bleaching (ASB; see methods 3.2) is reached (Fig. 1, NCC: net community calcification). The timing of the transition from states of net accretion to net erosion, however, is delayed when incorporating mitigation and adaptation factors into the projections. The maintenance of framework accretion until 2100 and beyond is only feasible under a lower emissions scenario (SSP2-4.5: CO_2_ emissions begin to fall mid-century, but do not reach net-zero by 2100) coupled with thermal adaptation above 0.5˚C. Although restoration initiatives increase reef accretion, and, along with thermal adaptation, enable vertical growth to keep pace with SLR (global mean sea-level rise (GMSLR) projections) for a prolonged duration, these benefits are rapidly lost with the onset of ASB. Only in the best-case scenarios (SSP2-4.5 and thermal adaptation > 0.5˚C), can Cheeca Rocks reef keep pace with SLR projections beyond 2100.

The vulnerability of reef persistence remains ultimately dependent on the rate and extent of climate change. However, increases in thermal tolerance of corals may delay the onset of mass mortalities in time for low emission scenarios to be implemented and management interventions to become effective.

### Carbonate budgets trajectories

The model suggests that the most severe declines in carbonate production occur when no thermal adaptation is incorporated into the projections. Under the SSP5-8.5 scenario, reef accretion ceases, on average, in 2069 (NCC =  − 0.04 ± 0.76 kg m^−2^ yr^−1^) but ranging from 2059 to 2079 depending largely on initial transect coral cover. The onset of net loss is postponed 13 years (~ 2082, ranging from 2069 to 2100) when CO_2_ emissions start declining mid-century under SSP2-4.5 (NCC =  − 0.002 ± 0.72 kg m^−2^ yr^−1^).

The deposition of carbonate by corals and its modelled trajectory under the different scenarios plays the biggest role in driving carbonate budgets at Cheeca Rocks^8,34^. While coral cover on offshore reefs along the Florida Keys is on average less than 5%^35^, Cheeca Rocks has maintained more than 25% coral cover for the past decade (Figure S1). The resilience of these inshore patch reef communities is attributed to the fluctuating environment they reside in^36^. The large shifts in temperature and pH have likely already caused some degree of adaptation in corals at Cheeca Rocks compared to other reefs in the Florida Keys^37^. Even after the two warmest summers on record, the site only experienced 3.7% decline in coral cover. Gintert et al. (2018) found evidence of acclimatization between the two consecutive years of bleaching (DHW = 7.7 in 2014 and DHW = 9.5 in 2015), however it remains unclear if the reef would still persist past a higher DHW threshold or after the onset of ASB, making it increasingly more difficult for corals to recover sufficiently between bleaching episodes.

Little is known about the capacity for reef-forming taxa to become more tolerant to ocean warming and marine heatwaves. There is, however, increasing evidence that responses of corals to thermal stress are changing with time and exposure^37,38^. Main adaptive processes include symbiont shuffling towards more heat tolerant genotypes^39,40^, physiological acclimatization (e.g., upregulation of heat shock proteins^41^), natural selection for heat tolerant genotypes resulting in shifts in prevalence of sensitive species to more tolerant ones^42^, and community shifts where sensitive species are replaced by tolerant ones^43^. The extent to which corals will adapt depends on these varying mechanisms and will vary accordingly in time and across geographical scales^33,44^. In the present study, no assumptions are made as to which of the various mechanisms, by which coral thermal tolerance increases, is at play.

When incorporating varying levels of thermal adaptation in the habitat persistence projections, the expected timing of the onset of ASB is delayed at different scales under the two greenhouse gas emission scenarios. If corals adapt to an additional 0.25˚C across transects at Cheeca Rocks, net carbonate accretion is projected to cease on average by 2074 under SSP5-8.5 (ranging from 2064 to 2083 depending on transects), 5 years later than in a scenario without thermal adaptation. Under a reduced emission scenario, on the other hand, net accretion is still occurring by 2100 (18 years later), albeit very low (NCC = 0.15 ± 0.72 kg m^−2^ yr^−1^). With the incorporation of an additional 0.5 and 1˚C adaptation, accretion cessation is pushed forward another 4 and 10 years (2078 and 2088) respectively under SSP5-8.5. By 2100, under the SSP2-4.5 scenario coupled with 0.5 and 1˚C adaptation, carbonate budget rates amount to 2.88 ± 1.96 and 6.41 ± 3.43 kg m^−2^ yr^−1^ respectively on average. Declines in modelled net accretion under SSP2-4.5 do not occur anymore once corals have adapted to more than 0.5 ˚C. With 2˚C adaptation, under SSP5-8.5, mean net accretion is still occurring by 2100 yet declines to 0.79 ± 1.29 kg m^−2^ yr^−1^.

Projected reduced net carbonate production is mainly driven by loss of coral cover due to the onset of ASB rather than the impacts of ocean warming and acidification on rates of calcification and bioerosion^8,34^. When the impacts of coral bleaching are excluded from our projections, modelled carbonate production rates under the SSP2-4.5 scenario increase on average by 25% in 2050 and 88% by 2100 (Table 1). Projections under SSP5-8.5 increase by 18% in 2050 and start decreasing again by 2070. Such scenarios, without bleaching, would only be feasible if the thermal adaptation of coral increased to the point where they would cease to be vulnerable to bleaching. The modelled projections suggest that corals would need to become tolerant to more than 0.5˚C under reduced CO_2_ emissions (SSP2-4.5) to push the onset of ASB beyond 2100. Under the SSP5-8.5 scenarios, more than 2˚C adaptation would be necessary. Although the impacts on calcification and bioerosion caused exclusively by increasing SST and ocean acidification appear to be modest compared to the impact of severe bleaching, their effects will likely be more noticeable on already degraded reefs. Once corals, and therefore their contribution to net production have declined past a certain point, bioerosion processes take over and dictate carbonate budgets trajectories, despite a relatively marginal/modest anticipated increase in bioerosion rates (Table 1, Figure S5). This is due to total bioerosion being predominantly represented by the contribution of parrotfish and sea urchins, which erode coral framework using mechanical processes that are not significantly affected by OA and SST. However, macro- and micro-bioerosion are likely to benefit not only from factors that impede coral calcification such as OA and eutrophication^15,45^ but also from increased availability of suitable substratum following coral mortality. Mass coral die-offs due to heatwaves have indeed been linked to increased abundances of bioeroding sponges^46^. The contribution of coralline algae to net budgets did not play a major role especially in the SSP5-8.5 scenario due to their sensitivity to the effects of OA. With regards to temperature, CCA appear more robust than corals and may initially act as a slight buffer to declines in net carbonate production following coral mortality. But this contribution will include less-stable forms of calcium carbonate that will generally not support upward reef growth and habitat characteristics otherwise provided by reef-building corals. Future conditions have been shown to favor ephemeral accretion by heterotrophic fast-growing organisms, undermining reef structural integrity and persistence^47^. Ultimately, the preservation of a structurally complex habitat will rely on the survival or reconstruction of coral communities.

**Table 1:** Mean percentage change in relative contribution of each process involved in the calculation of net carbonate budgets and mean change in net carbonate accretion.

Contributing parameters	Adaptation	SSP2-4.5			SSP5-8.5		
		2050	2075	2100	2050	2075	2100
Coral calcification	0˚C adaptation	15%	− 53%	− 82%	3%	− 72%	− 91%
	+ restoration	+ 36%	− 44%	− 79%	+ 21%	− 68%	− 90%
	0.25˚C adaptation	+ 23%	+ 12%	− 62%	+ 18	− 64%	− 90%
	+ restoration	+ 46%	+ 25	− 59%	+ 37%	− 59%	− 89%
	0.5˚C adaptation	+ 23%	+ 39%	− 3%	+ 20%	− 53%	− 88%
	+ restoration	+ 36%	+ 52%	+ 4%	+ 40%	− 46%	+ 86%
	1˚C adaptation	+ 23%	+ 45%	+ 75%	+ 20%	− 1%	− 80%
	+ restoration	+ 43%	+ 68%	+ 103%	+ 40%	+ 14%	− 77%
	2˚C adaptation	+ 23%	+ 45%	+ 75%	+ 20%	+ 29%	− 35%
	+ restoration	+ 43%	+ 68%	+ 103%	+ 40%	+ 50%	− 25%
	No Bleaching	+ 23%	+ 45%	+ 75%	+ 20%	+ 29%	+ 25%
	+ restoration	+ 43%	+ 68%	+ 103%	+ 40%	+ 50%	+ 35%
CCA calcification	All adaptation scenarios	+ 22%	+ 12%	− 6%	+ 14%	− 100%	− 100%
Macroborers (sponges)		+ 33%	+ 67%	+ 104%	+ 35%	+ 75%	+ 123%
Microborers		+ 53%	+ 88%	+ 96%	+ 76%	+ 160%	+ 230%
Grazing (urchin and parrotfish)		+ 0%	+ 0%	+ 0%	+ 0%	+ 0%	+ 0%
Net carbonate budget	0˚C adaptation	+ 14%	− 81%	− 122%	− 4%	+ 117%	− 151%
	+ restoration	+ 42%	− 70%	− 118%	+ 18%	− 112%	− 149%
	0.25˚C adaptation	+ 25%	+ 5%	− 96%	+ 15%	− 106%	− 149%
	+ restoration	+ 55%	+ 23%	− 91%	+ 40%	− 99%	− 148%
	0.5˚C adaptation	+ 25%	+ 41%	− 16%	+ 18%	− 91%	− 146%
	+ restoration	+ 52%	+ 73%	+ 5%	+ 45%	− 62%	− 145%
	1˚C adaptation	+ 25%	+ 50%	88%	+ 18%	− 22%	− 136%
	+ restoration	+ 52%	+ 81%	+ 126%	+ 45%	− 1%	− 133%
	2˚C adaptation	+ 25%	+ 50%	88%	+ 18%	+ 20%	− 76%
	+ restoration	+ 52%	+ 81%	+ 126%	+ 45%	+ 47%	− 63%
	No Bleaching	+ 25%	+ 50%	88%	+ 18%	+ 20%	+ 4%
	+ restoration	+ 52%	+ 81%	+ 126%	+ 45%	+ 47%	+ 29%

Percentage changes due to warming and OA in each greenhouse gas emission scenario are calculated for 2050, 2075 and 2100, relative to the year 2019. Percentage change in coral calcification contribution to budgets in 2050 and 2100 are also shown for different adaptation scenarios and with restoration.

### Reef accretion, SLR and accumulated height

**Figure 2:**
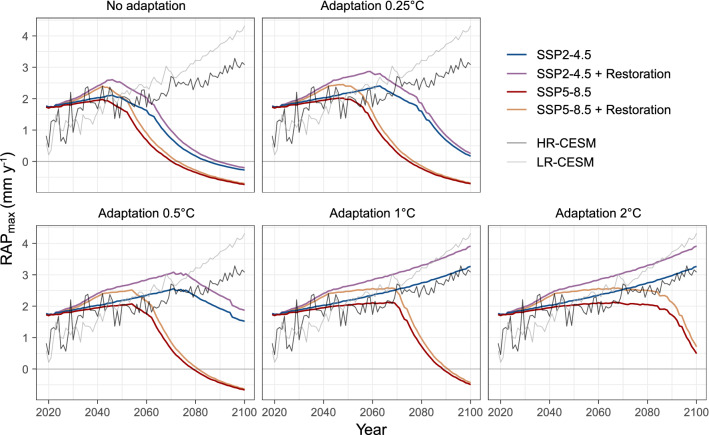
Mean potential reef accretion (RAP_max_ in mm yr^−1^) projections at Cheeca Rocks from 2019 to 2100 under the SSP2-4.5 (blue) and SSP5-8.5 (red) scenarios. Panels depict RAP_max_ rates in scenarios where no adaptation, 0.25, 0.5, 1 and 2˚C adaptation were incorporated. The purple and orange line represents vertical accretion when M:IR cover targets are incorporated in the carbonate budget projections for SSP2-4.5 and SSP5-8.5 respectively. Trends in reef accretion are compared to SLR projections using a low- and high-resolution version of CESM (LR-CESM in grey and HR-CESM in black) (van Westen et al., 2021). The CESM SLR time series are retained near Cheeca Rocks (spatial average over 24.5 N–25.5 N and 80 W–81 W).

The framework of many shallow coral reefs plays an important role in dissipating wave energy, acting as natural breakwaters and protecting the shoreline and coastal infrastructure from the threats of erosion and flooding^6,7^. The maintenance of this valuable ecosystem service, which is heavily influenced by reef carbonate deposition capacity and accretion potential, is therefore a major concern for coastal managers and restoration practitioners. Accordingly, evaluating the capacity of vertical reef growth to keep pace with projected SLR is a pressing matter for the development of timely flooding and erosion mitigation actions. Average RAP_max_ across the six transects at Cheeca Rocks was estimated to be 1.76 ± 1.05 mm yr^−1^ in 2019 which falls within range of previous measurements made in the Caribbean^3,8,13^, but is substantially higher than the average reef accretion potential throughout the Florida Keys^18^. By 2100, projected RAP_max_ rates in most scenarios have either decreased considerably or become negative (Fig. 2). Only under the SSP2-4.5 scenario combined with > 0.5˚C adaptation, does accretion persist, reaching on average 3.2 ± 1.68 mm yr^−1^ by 2100.

The projected total sea level rise from 2000 to 2100 at Cheeca Rocks ranges from 3.1 to 4.4 cm per decade (HR-CESM and LR-CESM, respectively), depending on the horizontal resolution of the CESM^48^. While accretion persistence varies between scenarios, depending on the level of incorporated thermal adaptation, all the RAP_max_ projections at Cheeca Rocks keep pace with both high- and low-resolution SLR projections until 2050 (Fig. 2). After 2050, the capacity to keep pace depends on CO_2_ mitigation and levels of adaptation. By 2100, under SSP5-8.5 scenarios, regardless of the level of adaptation, all reef accretion projections are lower than SLR projections. Even with the incorporation of 2˚C adaptation reef accretion becomes too slow by 2068. This implies that Cheeca Rocks will not be able to keep up with sea level rise, with possible consequences for shoreline integrity. This is particularly bleak as Cheeca Rocks is a site with relatively high coral cover and structurally complex reef habitat compared to the rest of the Florida reef. Under the more optimistic scenario where CO_2_ emissions start to decline mid-century (SSP2-4.5) and corals adapt to more than 0.5˚C (i.e., no bleaching occurs), reef accretion projections follow SLR projections from the high-resolution scenario relatively well (HR-CESM) (Fig. 2).

Accumulated height trajectories (Fig. 1, bottom panels) illustrate the evolution of reef height accumulated through time up to 2100. The reef appears to plateau once the onset of ASB is reached, before starting to decline or becoming locked into accretionary stasis. Under SSP5-8.5 with no thermal adaptation, the height of the reef has still not circled back to today’s height by 2100, but it is clearly heading in that direction (net erosion). Only scenarios under SSP2-4.5 with 0.5˚C or more adaptation enable the reef to continue growing vertically beyond 2100 and even then, it is only projected to grow on average by 19.6 cm from 2019 onwards, which is close to the predicted increases in water depth above these reefs (~ 25 cm for the period 2019–2100^48^).

### Impact of restoration

Restoration has historically been a key tool to reverse habitat loss, restore ecosystem functions and tip reef processes back into upward growth, but the extent to which this will be sufficient under future climates is uncertain. The NOAA’s Mission: Iconic Reefs (M:IR) restoration initiative (NOAA, 2021) is an ambitious multi-agency effort at seven important reefs along the Florida Reef Tract (including Cheeca Rocks) aimed at reestablishing coral cover to historic baselines and restoring reef function. Our results show that the incorporation of M:IR restoration targets into the projections at Cheeca Rocks results in higher net reef carbonate production (Table 1) and related accretion potential estimates (Fig. 2). Under SSP5-8.5, however, this only resulted in a small 2-years delay in the shift to negative budgets with 0, 0.25, 0.5 and 1˚C adaptation. Under SSP2-4.5 coupled with no adaptation, restoration interventions result in a 5-year delay in the shift to net framework loss. With 0.25˚C adaptation however, negative budgets are pushed forward beyond 2100 (Figure S6). While restored reef projections suggest that vertical accretion will not be sufficient to keep pace with projections for SLR under SSP5-8.5 (all levels of adaptation) and SSP2-4.5 (with 0, 0.25 and 0.5˚C adaptation levels), results attest to the potential of restoration (Fig. 2).

In both SSP scenarios, the incorporation of restoration interventions prolongs the time in which reef accretion projections can keep pace with HR-CESM SLR projections. For instance, under SSP2-4.5 with no adaptation, coral planting extends projection matching by 7 years. Under SSP5-8.5 coupled with 2˚C adaptation, RAP_max_ projections keep pace with SLR projections for an additional 17 years when restoration interventions are applied. Although global-scale action on climate change is critical for ensuring reef persistence, bold active restoration initiatives that incorporate stress hardening approaches to increase thermotolerance of corals^49^ may create the necessary bridge to sustain the reef structure long enough for a low-carbon economy to become effective.

### A tool for efficient local management

The tailored modelling approach presented here provides a helpful tool for managers in their attempts to prevent deleterious effects from climate change and SLR. In its present form, however, it solely applies to the reefs at Cheeca Rocks and few similar locations throughout the Florida Keys. The current modelling approach can be applied to many other reefs from diverse geographic regions as the input data (e.g. site-specific growth rates) is in some cases readily available and the spatial resolution of climatological data continues to improve.

Local mitigation actions have been shown to be essential to prevent reef functional collapse^34^. But decision makers are often limited by the lack of predictive tools that provide the necessary information for their specific reef to assess the impact that management initiatives such as coral and grazer introduction or seagrass beds restoration for carbon sequestration purposes, would have on habitat persistence in the context of projected global change and ongoing anthropogenic pressures. Site-specific evaluations of habitat persistence trajectories can be used to adequately design and improve locally tailored interventions and set appropriate targets to allow reef accretion to keep pace with SLR. Additionally, as shown in this study, projections can be used as a tool to assess the impact of planned mitigation strategies and evaluate the desired habitat restoration outcomes.

## Method considerations and conclusion

Some degree of assumptions and error must be accepted in projecting future carbonate trajectories. This modelling approach does not incorporate factors such as increased frequency and intensity of storms, the impacts of diseases (e.g., stony coral tissue loss disease) and decreasing water quality. For example, studies testing impacts of eutrophication on calcification and bioerosion at the species and community level have found that nutrient enrichment negatively affects coral physiology and ecosystem functioning^50,51^ while accelerating bioerosion^15,52^. Coastal runoff has also been shown to exacerbate OA and bleaching susceptibility^53^.

Additionally, thermal adaption by corals to recurring bleaching events is not fully explored in this model. A range of species-specific adaption responses are to be expected and should be incorporated in future modelling efforts. We do not, however, have sufficient data on adaptive capacity at this time, and lack a hierarchical understanding of how different species compare. Instead, we employ the extreme ASB endpoint because it is likely that most species will be affected when this threshold is reached. Additionally, we note that adaptation can be a gradual process and coral populations are unlikely to have already adapted to 2˚C at time zero. We investigate different degrees of adaptation (namely 0, 0.25, 0.5, 1 and 2˚C) to show the gradual shift in the ASB threshold (the tipping point when the reef will change dramatically) throughout time even after having reached such degrees of adaptation. Models could incorporate different assumed rates of population adaptation (e.g. 0.2 versus 0.5 °C per decade) and these could be applied to each species differently, potentially resulting in different trajectories than those observed herein. Further, it is likely that coral cover itself could alter these rates, as increasingly sparse and separated colonies will have diminishing chances of reproductive success.

Despite these limitations and potential future directions, this model serves as the framework for a tool to project site-specific capacities of coral reefs to maintain a positive carbonate budget and is the first to incorporate the impact of potential coral reef thermal adaptation in habitat persistence projections. The projected outcomes demonstrate how difficult it is, even for seemingly resilient reefs, to maintain their physical habitat in an increasingly hostile environment. Human interventions, however, may still be capable of ensuring reef persistence provided they are appropriately scaled to the local manifestations of global change.

## Materials and Methods

**Figure 3:**
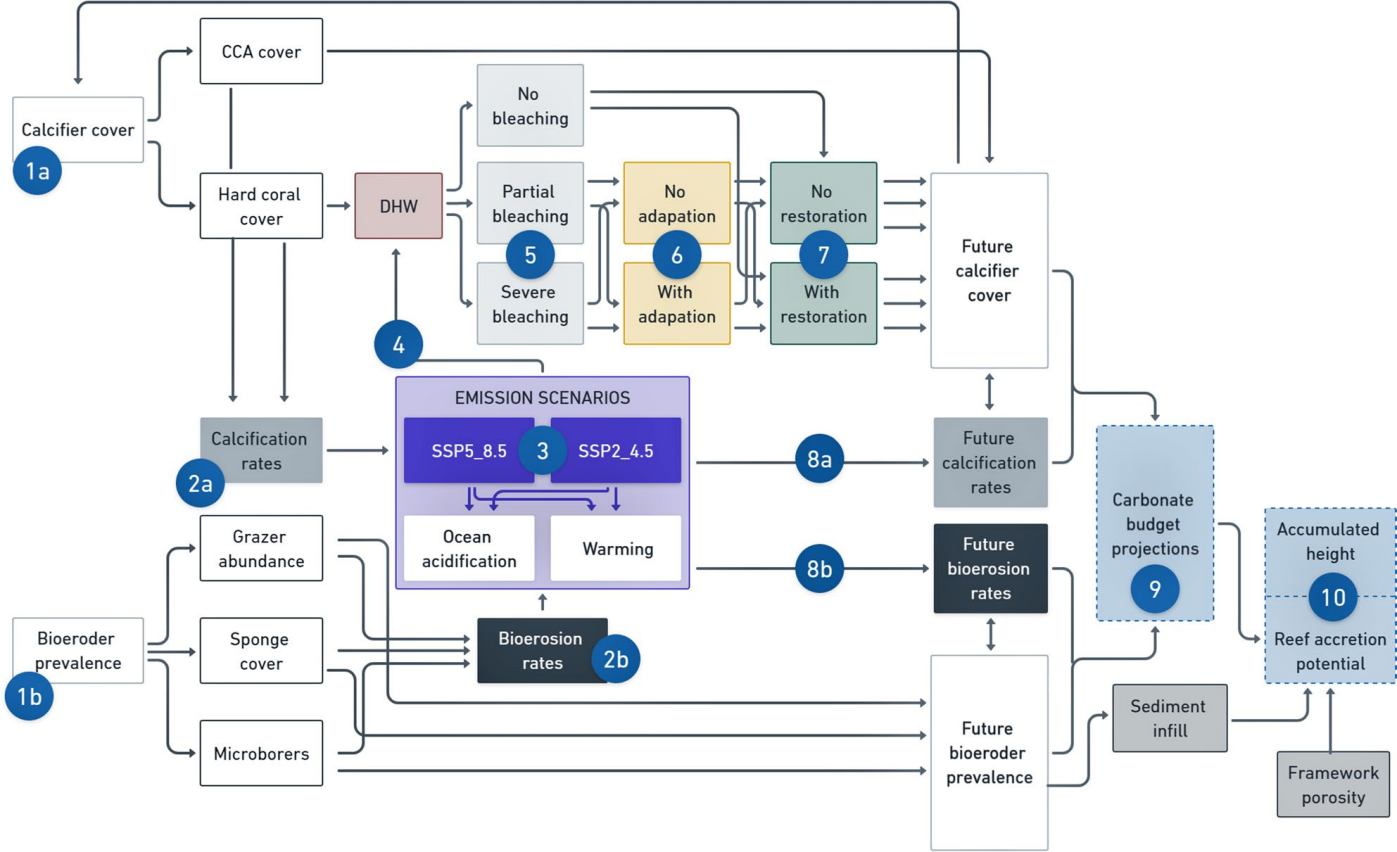
Diagram illustrating the structure of the habitat persistence algorithm for the projection of annual carbonate budget and reef accretion projections. Numbers 1 to 7 relate to the main parameters which are incorporated in the model and are described in detail in the methods section. CCA refers to calcifying coralline algae and DHW refers to degree heating weeks.

The habitat persistence model was created in the R software environment (R Core Team, 2020) and its structure is illustrated in Fig. 3. Model parameters were drawn from data collected at Cheeca Rocks in the last decade, published literature on Cheeca Rocks and Caribbean reefs, and from climate data from the Coupled Model Intercomparison Project Phase 6 (CMIP6^54^) new generation of climate models forced by the Shared Socioeconomic Pathways (SSPs^55,56^). Numbered portions in sections "Model inputs and components" and "Model outputs" below describe the main parameters utilized in the model and relate to the numbers in Fig. 3.

### Study site

Cheeca Rocks (24.897377° N, 80. 61,801° W) is a shallow inshore patch reef situated near Islamorada between 2 and 6 m depth. Six permanent 10 m transects have been surveyed nearly annually since 2012 as part of NOAA’s National Coral Reef Monitoring Program (NOAA Coral Program^57^). Surveys followed the *ReefBudget* approach^16^, a field-based census methodology to determine net carbonate production through a carbonate budget approach (see Figure S1). The latter are built based on the abundance of all major carbonate producing and eroding taxa and the rates at which these processes operate.

### Model inputs and components

#### Calcifier cover

The percent cover of calcifiers (corals, hydrocorals, and crustose coralline algae) were extracted from in situ visual surveys conducted along the six permanent 10 m line transects. Briefly, a 15 m weighted transect is draped over the reef framework and the cover (in cm) of all species of corals, types of substrates, as well as other major taxa such as crustose coralline algae, *Halimeda*, and sponges, are recorded below the entire transect line and following the full contour of the reef. The benthic composition of each major functional group is then converted to percent cover.

#### Bioeroder prevalence

The abundance, percent cover, and sizes for the major bioeroding taxa (parrotfish, excavating sponges, sea urchins) were extracted from 3 separate types of surveys. 1) Surface area of clionaid sponge cover is recorded using a quadrat on a 1 m wide belt transect (50 cm each side) along the main 10 m linear transection. "Results and discussion") The test diameter of every urchin species occurring on a 2 m wide belt transect (1 m each side) along the main 10 m linear transect is measured. 3) Parrotfish abundance, species, phase, and size class are recorded (according to the *ReefBudget* methodology) within 4 m wide and 30 m long belt transects (*n* = 10) across the site.

#### Calcification rates

Coral CaCO_3_ production rates were calculated based on the measured and projected cover for each species and species‐specific growth rates and skeletal density derived from published Caribbean data^16^. Similarly, carbonate production by crustose coralline algae (CCA) was calculated using cover and a standard calcification rate.

These default input rates were used in the present study with some rate substitutions when direct measurements carried out in the Florida Keys were available. Measurements of local mean calcification rates for *O. faveolata* (12.0 kg CaCO_3_ m^−2^ y^−1^), *Porites astreoides* (6.9 kg CaCO_3_ m^−2^ y^−1^), *Siderastrea siderea* (9.9 kg CaCO_3_ m^−2^ y^−1^) and crustose coralline algae (0.5 kg CaCO_3_ m^−2^ y^−1^) replaced default rates form the *Reefbudget* method^12,58,59^.

#### Bioerosion rates

Erosion by four bioeroding groups: (1) macroborers, (2) parrotfish, (3) urchins and (4) microborers was estimated following *ReefBudget* v2 methodology (Perry and Lange, 2019). 1) Macrobioerosion was calculated by multiplying the total surface area of bioeroding sponges (*Cliothosa delitrix*, *Cliona caribbaea*, *Cliona tenuis* and *Cliona varians*) at each transect by species-specific bioerosion rates and dividing that value by the total survey area. 2) Parrotfish bioerosion rates were calculated by multiplying the abundances of parrotfishes by the estimated species- and size-specific bite rates provided in the ReefBudget v2 methodology. 3) Urchin bioerosion rates are estimated using size frequency data of urchins (*Echinometra* spp., *Diadema* spp.), collected from the benthic surveys, and multiplied by the test-size-specific bioerosion rate. 4) Microerosion rates were calculated from the percent cover of available substrate in each transect and a literature-derived rate of − 0.240 kg CaCO_3_ m^2^yr^−1^.

#### Emission scenarios

**Figure 4:**
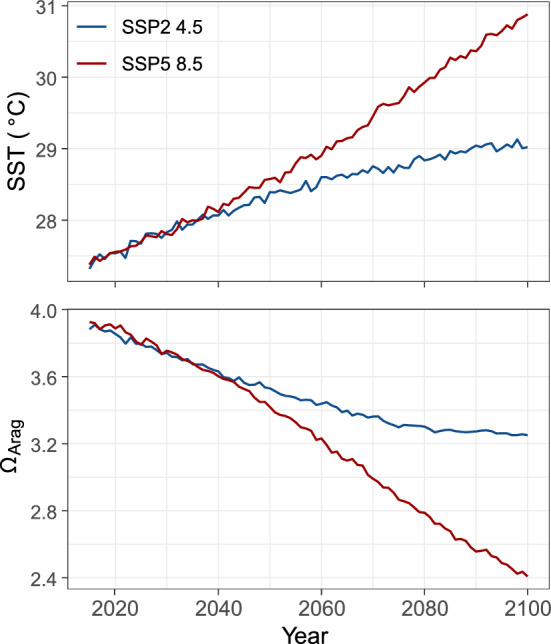
CMIP6 mean annual sea surface temperature (SST) and aragonite saturation state (Ω_Ar_) projections from 2019 to 2100 under two different emission scenarios. In blue; SSP2-4.5 and in red; SSP5-8.5.

Sea surface temperature (SST, variable name tos), sea surface salinity (SSS, variable name sos), surface Aqueous Partial Pressure of CO2 (variable name spco2) and pH native ocean model grid data were downloaded from https://esgfnode.llnl.gov/projects/cmip6 for all -models participating in—ScenarioMIP^56^ and providing SSP5-8.5 and SSP2-4.5 simulations. Using Climate Data Operators (https://code.mpimet.mpg.de/projects/cdo/), all data were re-gridded using bilinear interpolation to a 0.25º regular grid. Where multiple runs were available for a model, those were averaged first. Then a multi model ensemble was created for each variable. Ω_Ar_ was then computed from modelled variables using the package seacarb^60^ in the R software environment (R Core Team, 2020) (Fig. 4).

SSP5-8.5 is the pathway that represents current rates of emissions and emissions growth. It is considered a “worst case scenario” and it assumes there is no climate policy or that policy is not effective. SSP2-4.5 is a highly ambitious but still possible scenario. It is considered a “middle of the road” pathway.

#### Thermal stress and bleaching

Modelled SSTs were adjusted to measured values (2013–2021) at Cheeca Rocks by subtracting 2015–2021 August temperature average (30.48ºC) from the entire time series and adding the observed average August temperature (30.98ºC) from a Moored Autonomous pCO_2_ (MAPCO_2_) buoy that includes a conductivity-temperature sensor (Model SBE-16 plus v.2.2, Seabird Electronics). To predict heat stress caused by both the duration and intensity of marine heatwaves on coral reefs, we used the commonly used metric Degree Heating Weeks (DHWs). DHWs were computed as the sum of positive anomalies above the local observed bleaching threshold of 31.3ºC^37^ for each 3-month period. These were then converted to DHW by multiplying by 4.35^61^. Max DHW projections were then used to forecast annual bleaching. The onset of annual severe bleaching (ASB) conditions is described as the annual surpassing of > 8 DHWs accumulating during a 3-month period^26^. Eight DHWs is higher than the world average bleaching predictor^62^, but at this greater threshold, interspecies differences in sensitivity are covered and it is likely that most coral species will bleach^63^. Additionally, the last two annual bleaching events that took place at Cheeca Rocks occurred at 7.7 and 9.5 DHWs^37^.

#### Bleaching mortality

Since 2012, yearly coral reef image mosaics have been acquired over the six permanent transects to document detailed coral community information for over 4000 coral colonies through time. Species-specific trends at Cheeca Rocks under annual bleaching conditions were extracted from two documented bleaching events in 2014 and 2015 (Table 2) and incorporated in the carbonate budget projections to account for bleaching mortality on coral cover^37^. To account for intraspecies differences in bleaching-sensitivity, modest bleaching is incorporated when the site experiences DHWs between 4 and 8^64^ and is assumed to cause a third of the impact of ASB^43^. Since mortality is applied proportionally to the existing percent cover, coral cover drops to an infinitesimally small level. While zero is never reached, the calcification term becomes negligible in the carbonate budget, and the net result is entirely driven by the bioerosion terms.

**Table 2:** Percent cover reduction (relative to the percentage cover of the previous year) under annual severe bleaching (ASB, DHW > 8) and modest bleaching (MB, 4 < DHW > 8) for the eight main species found at Cheeca Rocks (based on Gintert et al*.,* 2018).

	Relative loss in cover per year	
	under ASB (> 8DHW)	under MB (> 4 and < 8 DHW)
*Orbicella faveolata*	6.80%	2.30%
*Orbicella annularis*	4.50%	1.50%
*Siderastrea siderea*	1.70%	0.60%
*Porites astreoides*	11%	3.70%
*Porites porites*	11%	3.70%
*Colpophyllia natans*	7.10%	2.40%
*Montastraea cavernosa*	12.50%	4.20%

#### Adaptation

The potential effects of adaptation are explored by increasing the temperature stress corals can experience before ASB; this is modelled by increasing the bleaching threshold by 0.25, 0.5, 1 and 2 °C (See UNEP report^65^). This resulted in 4 additional DHW projections for each greenhouse gas emission scenarios. Adaptation scenarios are applied to all coral species equally.

#### Restoration targets

Restoration targets specific to the Cheeca Rocks site were obtained from NOAA’s Mission: Iconic Reefs restoration project (NOAA, 2021). M:IR restoration goals are to increase coral cover by a total of 9% over 2 restoration phases spanning over 20 years. The phase 1 and phase 2 M:IR scenarios are based on target increases in the cover of *Orbicella* spp.*, Montastraea cavernosa*, brain coral (*Diploria labyrinthiformis* and *Colpophyllia natans*) and other stony coral species (*Siderastrea siderea* and *Porites astreoides*) (Table 3). Planting is scheduled to take place over 10 consecutive years in each phase.

**Table 3:** Percent increases in absolute coral cover in patch reef habitat at Cheeca Rocks based on the targets for Mission: Iconic Reefs used to project the impacts of restoration on habitat persistence (NOAA 2021).

*Coral taxon*	Target increase
Orbicella spp.	1.65%
Brain	1.15%
Total	2.80%
Orbicella spp.	3.35%
Brain	2.35%
Other small stony	0.50%
Total	6.20%

The aim is to increase coral cover by a total of 9% over 2 restoration phases spanning over 20 years. Phase 1 consists in increasing absolute coral cover by a total of 2.8% over approximately 10 years starting in 2022 and phase 2 involves increasing cover by an additional 6.2% in the following 10 years.

#### Future calcification and bioerosion rates

To account for changes in net calcification and bioerosion in response to warming and OA, data were compiled from the literature to build species-specific calcification (Fig. 3, 8a) and bioerosion (Fig. 3, 8b) response curves (Figure S2 and S3). We focused on experiments involving Caribbean coral species that were present in the 2019 Cheeca Rocks benthic surveys as well as experiments that manipulated CO_2_ and/or temperature (Table S1). Studies were not considered if there were insufficient carbonate chemistry data to calculate Ω_Ar_. Several less-studied species (i.e. *Agaricia* spp., *Madracis decactis*, *Millepora complanata* and *Mycetophyllia* spp.) did not have enough data points to build a response curve to OA and temperature. In those cases, we used the response curves of morphologically similar corals. In addition to hermatypic coral species, response curves were constructed for crustose coralline algae, macroboring clionid sponges and microborers (Figure S2 and Table S3). Effects of OA and warming on calcification and bioerosion rates of reef-forming taxa were included in the projections as an additive^31^, not synergistic, interaction representing a potentially conservative prediction for the impacts of global change on these species.

### Model outputs

#### Carbonate budget projections

Reef carbonate budget analyses were performed by combining the gross carbonate production standardized to transect-specific reef rugosity and bioerosion data to estimate annual mean net community calcification (kg CaCO_3_ m^−2^ y^−1^) (following Perry et al., 2012). The projected annual cumulative sum of carbonate production by all calcifiers and erosion by the four bioeroder groups on each transect was estimated using projected percent cover and OA and SST altered calcification and bioerosion rates. Sediment dissolution was also incorporated in the carbonate budget projections based on sand cover and sand dissolution rates as a function of Ω_Ar_ sourced from literature (Figure S4). To account for coral growth, 1% annual baseline growth was incorporated based on yearly average increases in coral cover at Cheeca Rocks in non-bleaching years (Figure S1). During years where bleaching is predicted to take place, this baseline growth parameter declines to 0.5%. Output included annual mean net community calcification (NCC) rates for each transect from 2019 to 2100 under the two emission scenarios, across a range of thermal adaptation states and after incorporation of restoration targets.

#### Reef accretion potential and accumulated height

Annual net reef carbonate production rates were converted to estimates of maximum vertical reef accretion potential (RAP_max_ in mm yr^−1^) following the method established by Kinsey et al. (1985) and Perry et al*.* (2018), based on carbonate density and framework porosity. No physical or storm-driven episodic removal of framework is accounted for in these calculations, and therefore RAP_max_ estimates are considered a maximum upward growth potential. To account for the incorporation of bioerosion-derived sediment back into the reef structure, and thus as an addition to the reef accretion rate metric, corrections for sediment infill were included as follow:

1 $$RAP_{{\max }} = \frac{{carbonate\;production\;rate + sediment\;in\;fill}}{{\rho \left( {1 - porosity} \right)}}$$

where sediment infill includes 25% of the sediment produced by parrotfish and 50% of the sediment produced by other bioeroding organisms^13^, ρ represents the CaCO_3_ density of 2.89 g cm^−3^ ^66^ and framework porosity for head and massive coral dominated assemblages such as Cheeca Rocks is estimated to be ~ 30% (but see supplements for RAP_max_ calculation considerations).

To visualize projected change in reef height through time, accumulated height in mm for any given year was calculated as the sum of RAP_max_ from previous years and the current year:

2 $$Accumulated\;height\left[ i \right] = RAP_{{\max \left[ {i - 1} \right]}} + RAP_{{\max \left[ i \right]}}$$

Finally, to examine if reef vertical growth at Cheeca Rocks has the capacity to keep pace with SLR, RAP_max_ rates were compared to global mean sea-level rise (GMSLR) projections in two versions of the Community Earth System Model (CESM). According to the 6th IPCC report, global mean sea level (GMSL) will likely rise 1 m above 1900 levels under the high emission SSP5-8.5 scenario in 2100. However, local mesoscale processes, such as ocean eddies, can cause deviations from this GMSL change^48^. This is highly relevant for the Florida Keys region because of its proximity to the Gulf Stream^67^. RAP_max_ at Cheeca Rocks were compared to a low-resolution version of CESM (LR-CESM) which has an ocean component with a 100-km horizontal resolution that cannot generate mesoscale ocean eddies, and to a high-resolution version of CESM (HR-CESM) which has an ocean component with a 10-km (0.1˚) horizontal resolution, capable of capturing the development and interaction of mesoscale ocean eddies^48^. We retained the regional SLR near Cheeca Rocks (spatial average over 24.5 N–25.5 N and 80 W–81 W) from the CESM output, the regional SLR consist of sterodynamic sea-level changes and freshwater input (i.e. melt from Antarctica, Greenland and glaciers).

## Supplementary Information

Supplementary Information.

## Data Availability

The datasets generated during and/or analyzed during the current study are available from the corresponding author on request.
