# Supplementary material for: Restoration and coral adaptation delay, but do not prevent, climate-driven reef framework erosion of an inshore site in the Florida Keys
Source: Sci Rep. 2023 Jan 5;13:258. doi: 10.1038/s41598-022-26930-4 (PMC9816163; doi:10.1038/s41598-022-26930-4)
Supplement: Supplementary file 1 — Supplementary Information. [file 41598_2022_26930_MOESM1_ESM.pdf]

Supplementary Materials

Restoration and coral adaptation delay, but do not prevent, climate-driven reef framework erosion of an inshore site in the Florida Keys.

Alice E. Webb[1,2]*, Ian C. Enochs[1], Ruben van Hooidonk[1,2], René M. van Westen[3], Nicole Besemer[1,2], Graham Kolodziej[1,2], T. Shay Viehman[4], Derek P. Manzello[5]

[1]*Ocean Chemistry and Ecosystems Division, NOAA Atlantic Oceanographic and Meteorological Laboratory, 4301 Rickenbacker Causeway, Miami, FL 33149, USA*

[2] *Cooperative Institute for Marine and Atmospheric Studies, Rosenstiel School of Marine and Atmospheric Science, University of Miami, 4600 Rickenbacker Causeway, Miami, FL 33149, USA*

[3]*Institute for Marine and Atmospheric Research Utrecht, Utrecht University, Princetonplein 5, 3584 CC Utrecht, Netherlands.*

[4]*National Centers for Coastal Ocean Science, NOAA National Ocean Service, 101 Pivers Island Rd, Beaufort, NC, 28516 USA*

[5]*Coral Reef Watch, Center for Satellite Applications and Research, NOAA Satellite Oceanography and Climatology Division, College Park, MD 20740*

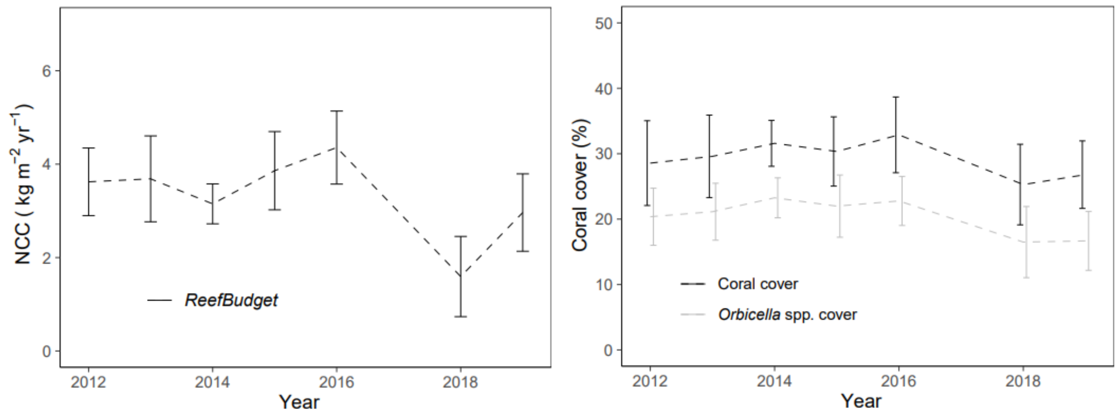

Figure S1. Carbonate budget rates (in kg m^{-2} yr^{-1}) and percent coral cover at Cheeca Rocks from 2012 to 2019. In the right panel, percent cover for all corals is depicted in black while the percent cover of the *Orbicella* species complex is illustrated in grey. Data was not collected in 2017 which was the year when hurricane Irma hit the Keys. The decrease in coral cover is likely linked to the hurricane and to tissue loss disease.

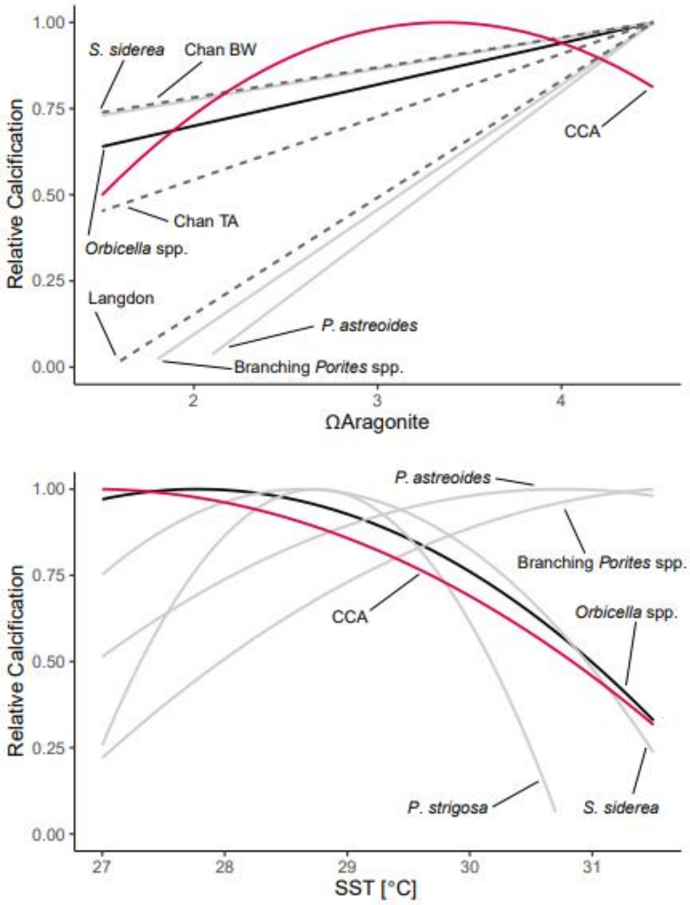

Figure S2. Main coral calcification response curves to aragonite saturation state (Ω_{Ar}) in the upper panel and to sea surface temperature (SST) in the lower panel. The data source utilized to build these response curves can be found in Table S1. In the upper panel, the dashed lines were added for comparison and represent response curves describe in Langdon et al. (2005)[1] and curves described in Chan and Conolly (2013)[2] using both the buoyant weight (BW) and the alkalinity anomaly (TA) techniques. CCA response curve is depicted in pink.

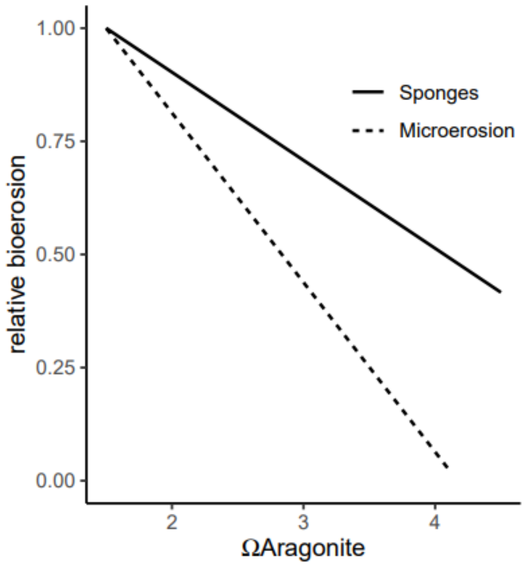

Figure S3. Relationship between seawater aragonite saturation state (Ω_{Ar}) and sponge bioerosion rates and coral microbioerosion rates. The data source utilized to build these response curves can be found in Table S1.

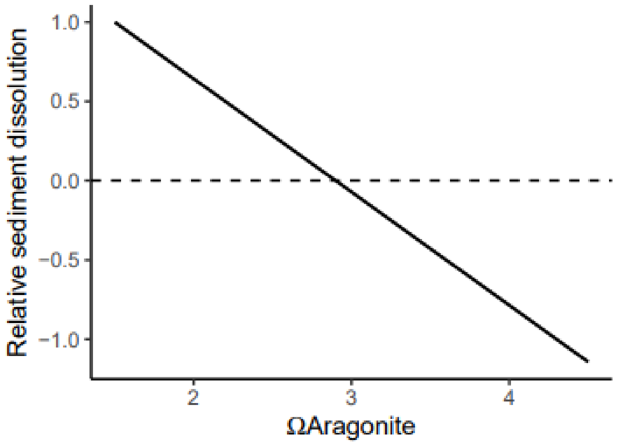

Figure S4. Relationship between seawater aragonite saturation state (Ωar) and reef carbonate precipitation/dissolution rates. The data source utilized (Table S1) to build this response curve come from Eyre et al. 2018[3].

Table S1. References for response curves in Figure S1, S2, and S3

Group	Stressor	Main data source
Coral calcification	OA	Jury et al. 2010[4]; Horvath et al. 2016[5]; Okazaki et al. 2017[6]; Bove et al. 2019[7]; Aichelman et al. 2020[8]
	SST	Jury et al. 2010; Grottoli et al. 2014[9]; Horvath et al. 2016[5]; Okazaki et al. 2017[6]; Bove et al. 2019[7]; Aichelman et al. 2020
CCA calcification	OA	Comeau et al. 2014a[10]; Comeau et al. 2014b[11]
	SST	Comeau et al. 2014a[10]; Comeau et al. 2016a[12] and 2016b; Johnson and Carpenter 2012[13]; Tanaka et al. 2017[14] and 2016; Vasquez et al. 2015[15]
Sponge bioerosion	OA	Fang et al. 2013[16]; Morris et al. Pers com; Wisshak et al. 2013[17]; Webb et al. 2017[18]
Micro bioerosion	OA	Enochs and Morris Pers com, Reyes-Nivia et al. 2013[19]; Tribollet et al. 2019[20]
Sand dissolution	OA	Cyronak et al. 2013[21]; Trnovsky 2016; Yates and Halley, 2003[22]; Andersson et al. 2007[23]; Fink et al. 2017[24]; Eyre et al. 2018[3]

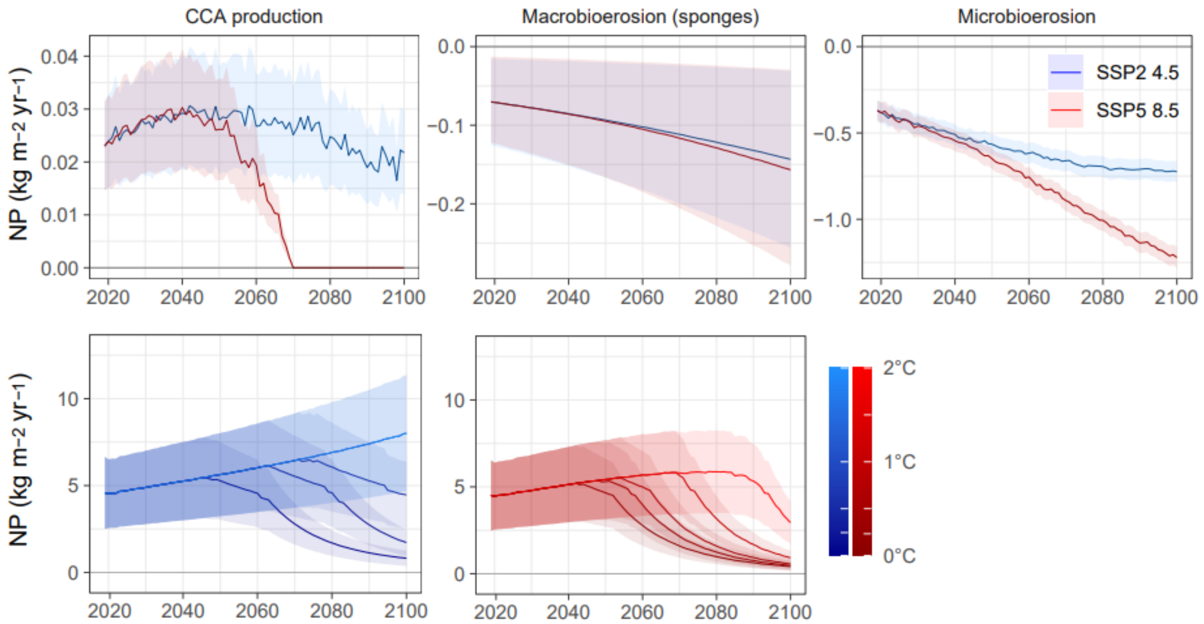

Figure S5. Mean relative contribution to net carbonate budget trajectories under the SSP2 4.5 (in blue) and SSP5 8.5 (in red) emission scenarios. The upper panels show relative contribution of secondary calcification (calcifying coralline algae production), sponge bioerosion and microbioerosion. The lower panels depict net carbonate production by corals where 0. 0.25, 0.5, 1 and 2 ˚C adaptation levels were incorporated in the projections. Shaded areas represent standard deviation from the mean of all six transects.

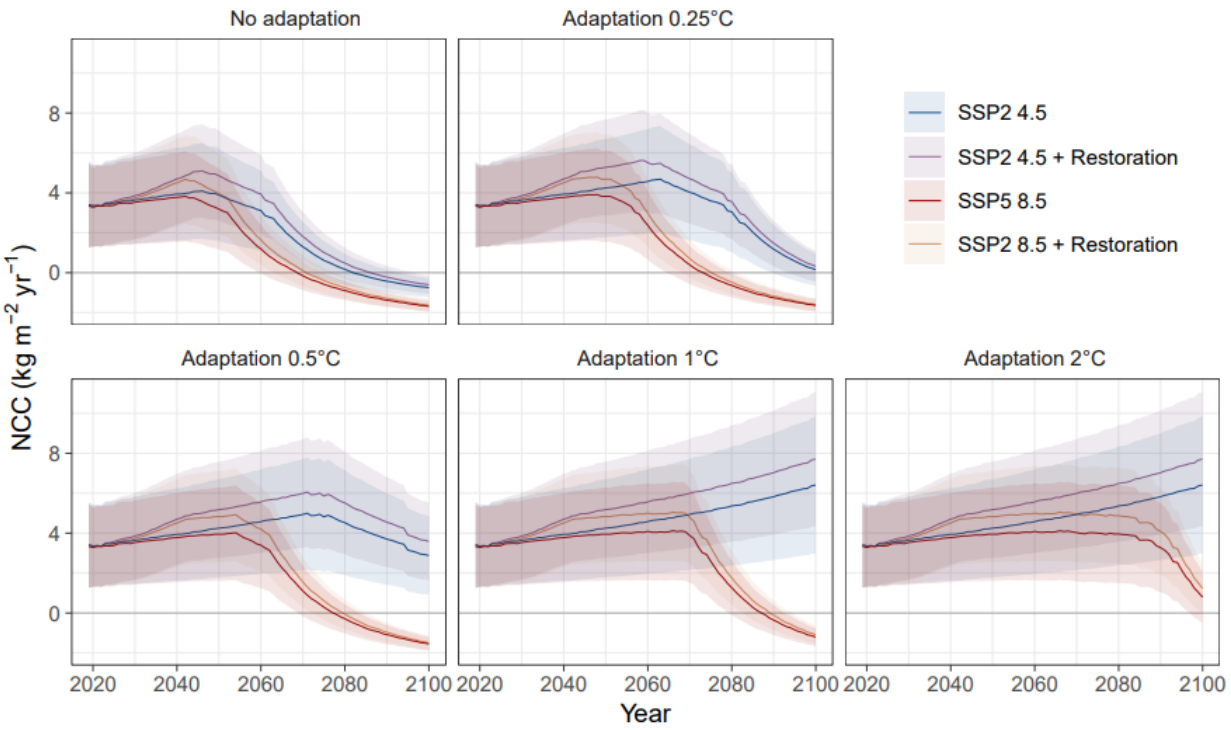

Figure S6. Trajectories of net carbonate budgets at Cheeca Rocks from 2019 to 2100 under the SSP2 4.5 (blue) and SSP5 8.5 (red) scenarios. Panels depict net community calcification (NCC) rates in scenarios where 0. 0.25, 0.5, 1 and 2 ˚C adaptation levels were incorporated in the projections. The green and orange lines illustrate net carbonate budgets trajectories when M:IR restoration cover targets are incorporated into the projections under SSP2 4.5 and SSP5 8.5 respectively.

RAP_{max} calculation considerations

Converting carbonate production rates to accretion potential rates is complicated25 and without true measure of reef porosity at Cheeca Rocks, caveats arise in their quantification. Here, we assume a porosity of 30% based on Perry et al. (2018)26 although Toth et al. (2022)27 calculated average porosity in the Florida Keys to be 59% for massive framework. However, sampled cores did not include patch reef habitat and large temporal variability was measured throughout the cores. Additionally, their method did not allow for distinction between true framework porosity from framework infilled with sediment due to the latter getting blown out during the coring process. RAPmax estimates are already considered optimistic because the quantification method does not consider physical or storm-driven episodic removal of framework28 and therefore in this study we conservatively assumed 30% porosity for Cheeca Rocks.

References

1. Langdon, C. & Atkinson, M. J. Effect of elevated pCO2on photosynthesis and calcification of corals and interactions with seasonal change in temperature/ irradiance and nutrient enrichment. *J. Geophys. Res. C Ocean.* **110,** 1–16 (2005).

2. Chan, N. C. S. & Connolly, S. R. Sensitivity of coral calcification to ocean acidification: A meta-analysis. *Glob. Chang. Biol.* **19,** 282–290 (2013).

3. Eyre, B. D. *et al.* Coral reefs will transition to net dissolving before end of century. **911,** 908–911 (2018).

4. Jury, C. P., Whitehead, R. F. & Szmant, A. M. Effects of variations in carbonate chemistry on the calcification rates of Madracis auretenra (= Madracis mirabilis sensu Wells, 1973): Bicarbonate concentrations best predict calcification rates. *Glob. Chang. Biol.* **16,** 1632–1644 (2010).

5. Horvath, K. M. *et al.* Next-century ocean acidification and warming both reduce calcification rate , but only acidification (not warming) alters skeletal morphology of reef-building coral Siderastrea siderea.(2016)

6. Okazaki, R. R. *et al.* Species-specific responses to climate change and community composition determine future calcification rates of Florida Keys reefs. *Glob. Chang. Biol.* **23,** 1023–1035 (2017).

7. Bove, C. B. *et al.* Common Caribbean corals exhibit highly variable responses to future acidification and warming. *Proc. R. Soc. B Biol. Sci.* **286,** (2019).

8. Aichelman, H. E., *et al.* Exposure duration modulates the response of Caribbean corals to global change stressors. Limnol. Oceanogr. **66**, 3100-3115, (2021).

9. Grottoli, A. G. *et al.* The cumulative impact of annual coral bleaching can turn some coral species winners into losers. *Glob. Chang. Biol.* **20,** 3823–3833 (2014).

10. Comeau, S., Carpenter, R. C. & Edmunds, P. J. Effects of irradiance on the response of the coral Acropora pulchra and the calcifying alga Hydrolithon reinboldii to temperature elevation and ocean acidification. *J. Exp. Mar. Bio. Ecol.* **453,** 28–35 (2014).

11. Comeau, S., Edmunds, P. J., Spindel, N. B. & Carpenter, R. C. Fast coral reef calcifiers are more sensitive to ocean acidification in short-term laboratory incubations. *Limnol. Oceanogr.* **59,** 1081–1091 (2014).

12. Comeau, S., Carpenter, R. C., Lantz, C. A. & Edmunds, P. J. Parameterization of the response of calcification to temperature and pCO2 in the coral Acropora pulchra and the alga Lithophyllum kotschyanum. *Coral Reefs* **35,** 929–939 (2016).

13. Johnson, M. D. & Carpenter, R. C. Ocean acidification and warming decrease calcification in the crustose coralline alga Hydrolithon onkodes and increase susceptibility to grazing. *J. Exp. Mar. Bio. Ecol.* **434–435,** 94–101 (2012).

14. Tanaka, Y., Suzuki, A. & Sakai, K. Effects of elevated seawater temperature and phosphate enrichment on the crustose coralline alga Porolithon onkodes (Rhodophyta).

Phycol. Res. **65,** 51–57 (2017).

15. Vásquez-Elizondo, R. M. & Enríquez, S. Coralline algal physiology is more adversely affected by elevated temperature than reduced pH. *Sci. Rep.* **6,** 1–14 (2016).

16. Fang, J. K. H. *et al.* Sponge biomass and bioerosion rates increase under ocean warming and acidification. *Glob. Chang. Biol.* **19,** 3581–3591 (2013).

17. Wisshak, M., Schönberg, C. H. L., Form, A. & Freiwald, A. Effects of ocean acidification and global warming on reef bioerosion-lessons from a clionaid sponge. *Aquat. Biol.* **19,** 111–127 (2013).

18. Webb, A. E. *et al.* Combined effects of experimental acidification and eutrophication on reef sponge bioerosion rates. *Front. Mar. Sci.* **4,** 311 (2017).

19. Reyes-Nivia, C., Diaz-Pulido, G., Kline, D., Guldberg, O. H. & Dove, S. Ocean acidification and warming scenarios increase microbioerosion of coral skeletons. *Glob. Chang. Biol.* **19,** 1919–1929 (2013).

20. Tribollet, A., Chauvin, A. & Cuet, P. Carbonate dissolution by reef microbial borers: a biogeological process producing alkalinity under different pCO 2 conditions. *Facies* **65,** 1–10 (2019).

21. Cyronak, T., Santos, I. R., McMahon, A. & Eyre, B. D. Carbon cycling hysteresis in permeable carbonate sands over a diel cycle: Implications for ocean acidificationn. *Limnol. Oceanogr.* **58,** 131–143 (2013).

22. Yates, K. K. & Halley, R. B. Measuring coral reef community metabolism using new benthic chamber technology. *Coral Reefs* **22,** 247–255 (2003).

23. Andersson, A. J., Bates, N. R. & Mackenzie, F. T. Dissolution of carbonate sediments under rising pCO2 and ocean acidification: Observations from Devil's Hole, Bermuda. *Aquat. Geochemistry* **13,** 237–264 (2007).

24. Fink, A. *et al.* Ocean Acidification Changes Abiotic Processes but Not Biotic Processes in Coral Reef Sediments. **4,** 1–13 (2017).

25. Hubbard, D. K., Miller, A. I. & Scaturo, D. Production and cycling of calcium carbonate in a shelf-edge reef system (St. Croix, U.S. Virgin Islands): applications to the nature of reef systems in the fossil record, *J. Sediment. Petrol*. **60**, 335–360. (1990)

26. Perry, C. T. *et al.* Loss of coral reef growth capacity to track future increases in sea level. *Nature* **558,** 396–400 (2018).

27. Toth, L. T., Courtney, T. A., Colella, M. A., Kupfner, S. A. & Robert, J. The past , present , and future of coral reef growth in the Florida Keys. 5294–5309 (2022). doi:10.1111/gcb.16295

28. Perry, C. T. *et al.* Caribbean-wide decline in carbonate production threatens coral reef growth. *Nat. Commun.* **4,** 1402–1407 (2013).
